# Distribution of regional lymph nodes metastasis in 870 cases of nasopharyngeal carcinoma and the suggestions for individualized elective prophylactic neck irradiation with intensity‐modulated radiotherapy

**DOI:** 10.1002/cam4.6723

**Published:** 2023-12-29

**Authors:** Lei Wang, Zheng Wu, Qian He, Yiting Li, Subin Wang, Feiping Li, Hui Wang, Wenhui Li, Yaqian Han

**Affiliations:** ^1^ Department of Radiotherapy Peking University Cancer Hospital Yunnan The Third Affiliated Hospital of Kunming Medical University Yunnan Cancer Hospital, Yunnan Cancer Center, Kunming Yunnan P.R. China; ^2^ Department of Radiation Oncology Hunan Cancer Hospital & the Affiliated Cancer Hospital of Xiangya School of Medicine Central South University Changsha Hunan P.R. China; ^3^ Department of Imaging, Hunan Cancer Hospital & the Affiliated Cancer Hospital of Xiangya School of Medicine Central South University Changsha Hunan P.R. China

**Keywords:** elective prophylactic neck irradiation, intensity‐modulated radiotherapy, lymph nodes distribution, nasopharyngeal carcinoma

## Abstract

**Purpose:**

To explore the feasibility of individualized elective prophylactic neck irradiation (iEPNI) for optimizing current approach by investigating metastatic lymph nodes (LNs) distribution in nasopharyngeal carcinoma (NPC).

**Materials and Methods:**

Records of 870 NPC patients without distant metastasis in Hunan Cancer Hospital from January 2019 to December 2019 were reviewed. LNs' locations were identified based on the 2013 guidelines. The intra‐regional lymphatic drainage (IRLD) areas included Station 1st (level VIIa and II), Station 2nd (level III and Va), and Station 3rd (level IV, Vb, and Vc). Other levels were categorized as extra‐regional areas.

**Results:**

Among the 870 patients, 94.5% cases exhibited LNs metastasis, including unilateral metastasis in 198 patients and bilateral metastasis in 624 patients. In the whole cohort, the most common involved IRLD areas were level IIb (87.1%), VIIa (80.0%), IIa (61.8%), Va (30.6%), IV (21.4%), Vb (8.9%), and Vc (1.1%). Besides, rates of LNs metastasis in Station 1st, 2nd, and 3rd were 94.3%, 61.1%, and 22.9%, respectively. Only four patients (4, 0.5%) revealed skipping metastasis among the three stations.

**Conclusions:**

Lymph node metastasis follows an organized pattern from Station 1st to 3rd with scarce skipping metastasis. A potential iEPNI strategy of prophylactic neck irradiation to the ipsilateral latter node‐negative station appears promising in NPC patients. Further prospective investigations are warranted to validate the approach.

## 1 INTRODUCTION

Nasopharyngeal carcinoma (NPC) is prevalent in southeast Asia but is rarely seen in western countries.^1^ Radiotherapy is the primary treatment modality, and combination of chemotherapy, including induction chemotherapy, concurrent chemotherapy or adjuvant chemotherapy, and radiotherapy, is recommended in both endemic and non‐endemic NPC.^2^, ^3^, ^4^ Besides, patients with NPC have achieved excellent prognosis with 5‐year overall survival of 80%.^5^, ^6^, ^7^

Nasopharyngeal carcinoma has a high incidence of lymph nodes (LNs) metastasis due to a well‐developed lymphatic network in the nasopharynx.^8^ A meta‐analysis has shown that 85% of Chinese NPC patients displayed LNs involvement at the time of diagnosis,^9^ and a retrospective analysis reported that approximately 30% of neck‐negative NPC patients subsequently developed LNs involvement without neck irradiation.^10^ Thus, prophylactic neck irradiation (PNI) is recommended for the treatment of NPC. However, traditional bilateral whole‐neck irradiation (WNI) often results in severe late toxicities, including hypothyroidism,^11^ subcutaneous fibrosis,^12^ and esophageal stenosis.^13^ Therefore, optimizing PNI for NPC has been widely investigated, aiming to reduce toxicities without impairing prognosis. The study by Ou et al. compared upper‐neck irradiation (UNI) of level II, III, and Va with WNI in NPC with retropharyngeal LNs (RLNs) only, and concluded that UNI was not inferior to WNI (5‐year nodal recurrence‐free survival, 89.3% vs. 100%, *P*=0.185).^14^ Furthermore, a recent prospective clinical trial confirmed these results with 3‐year regional relapse‐free survival (RRFS) of 97.7% in the UNI group and 96.3% in the WNI group (*P*_non‐inferiority_<0.0001) in NPC with N0‐1 disease.^15^ A recent meta‐analysis conducted by Felice et al. also supported the potential role of UNI compared of WNI in N0‐1 NPC in terms of treatment outcomes,^16^ and Felice et al. observed minimal dosimetric advantage of IMPT (Intensity Modulated Proton Therapy) plan compared with IMRT (Intensity‐Modulated Radiotherapy) plan in a N1‐stage NPC patient.^17^ Therefore, a consensus for UNI in node‐negative or only RLN‐positive NPC has been reached.^18^ However, based on this consensus, there are still approximately 70% of patients treated with WNI, since patients with only RLN‐positive or N0‐stage disease constitute around 30% of all NPC cases.^8^, ^19^

Multiple investigations have revealed an orderly spread pattern of LNs from the upper to the lower neck with rare skipping metastasis in NPC.^19^, ^20^, ^21^ Even there is LN metastasis in level II, skipping LN involvement in level IV, Vb, and Vc is still uncommon when no positive LNs are observed in level III and Va.^19^, ^20^, ^21^, ^22^ Thus, further individualized elective PNI (iEPNI) rather than UNI or WNI may be required. To address this issue, we investigated the LNs distribution patterns in non‐distant metastatic NPC patients based on the 2013 guidelines,^23^ in order to provide clinicians with a clue of iEPNI strategy.

## 2 MATERIALS AND METHODS

### 2.1 Patients

Magnetic resonance imaging (MRI) documents of newly diagnosed NPC patients between January 2019 and December 2019 in the Hunan Cancer Hospital were reviewed. The inclusion criteria included: (1) confirmed pathological diagnosis as NPC; (2) non‐distant metastatic disease; (3) available pretreatment MRI documents of the nasopharynx and neck; and (4) no prior antitumor treatment. Patients who previously underwent nasopharyngeal mass or neck LNs resection were excluded. Staging was performed using the 8th TNM staging system.^24^

### 2.2 MRI scan

All patients underwent a pretreatment MRI scan from the middle of the temporal lobe to the thoracic entrance, covering the nasopharynx and neck.^25^ The slice thickness was set to 5 mm with an inter‐slice gap of 0.5 mm. Axial T1‐weighted imaging (T1WI), T2‐weighted imaging (T2WI), and diffusion‐weighted imaging were obtained before a gadopentetate dimeglumine (Gd‐DTPA) injection. Following an injection with Gd‐DTPA, T1WI fat suppression sequence scans in the axial, coronal, and sagittal directions were conducted.

### 2.3 Image assessment

All MRI documents were reviewed by a radiologist and a radiation oncologist independently, and disagreements were resolved by discussion. The criteria for LNs metastasis were as follows: (1) cervical LNs with a minimum axial diameter (MAD) ≥ 10 mm; (2) ≥3 cervical LNs in the same high‐risk region with at least one LN having a MAD ≥8 mm; (3) RLNs with a MAD ≥5 mm; (4) any visible LNs in the median retropharyngeal region; and (5) any LNs with central necrosis, edge ring enhancement, or extracapsular invasion.^18^

### 2.4 Definition of LNs metastasis stations

Lymph nodes location was identified according to the 2013 guidelines.^23^ We categorized the intra‐regional lymphatic drainage (IRLD) areas into three stations based on the distribution of LNs: Station 1st comprising level VIIa and II, Station 2nd comprising level III and Va, and Station 3rd comprising level IV, Vb, and Vc. Other lymphatic drainage levels were considered as extra‐regional areas. Skipping metastasis was defined as LNs metastasis in Station 2nd with no positive LNs in Station 1st, and LNs metastasis in Station 3rd with no positive LNs in Station 2nd.

## 3 RESULTS

### 3.1 Patients

A total of 870 patients were enrolled, and the baseline characteristics are listed in Table 1. Of the entire cohort, 632 (72.6%) patients were male and 447 (51.4%) patients were >50 years old. A total of 862 (99.1%) patients had non‐keratinizing carcinoma. More than half of the patients had T3‐4 disease (475/870, 66.1%), as well as N2‐3 disease (591/870, 67.9%). Of the 870 patients, there were 118 (13.5%) cases staged I–II and 752 (86.5%) cases staged III–IVA.

**TABLE 1:** Baseline characteristics of the 870 NPC patients.

Characteristics	Number of patients (%)
Gender	
Male	632 (72.6)
Female	238 (27.4)
Age (year)	
≤50	423 (48.6)
>50	447 (51.4)
Histology	
WHO I	8 (0.9)
WHO II–III	862 (99.1)
T stage	
T1	132 (15.2)
T2	163 (18.7)
T3	374 (43.0)
T4	201 (23.1)
N stage	
N0	48 (5.5)
N1	231 (26.6)
N2	391 (44.9)
N3	200 (23.0)
TNM stage	
I	15 (1.7)
II	103 (11.8)
III	391 (45.0)
IVA	361 (41.5)

Abbreviations: NPC, nasopharyngeal carcinoma; WHO, World Health Organization.

### 3.2 Distribution of metastatic LNs at each level

Among the 870 patients with NPC, the incidence of LNs metastasis was 822/870 (94.5%), including 198 cases of unilateral metastasis and 624 cases of bilateral metastasis. The distribution of metastatic LNs in each level are presented in Table 2. The most commonly involved IRLD areas ranked level IIb (87.1%), VIIa (80.0%), IIa (61.8%), Va (30.6%), IV (21.4%), Vb (8.9%), and Vc (1.1%). The metastatic rates of the extra‐regional areas were 0.2% for level Ia, 7.7% for level Ib, 0.1% for level VI, 5.6% for level VIIb, 5.5% for level VIII, 0.3% for level IX, and 0.2% for level X.

**TABLE 2:** Characteristics of LNs spread of the 870 NPC patients.

Variables	Unilateral (No. %)	Bilateral (No. %)	Total (No. %)
Level Ia	/	/	2 (0.2)
Level Ib	47 (5.4)	20 (2.3)	67 (7.7)
Level IIa	313 (40.0)	225 (25.9)	538 (61.8)
Level IIb	268 (30.8)	490 (56.3)	758 (87.1)
Level II	243 (27.9)	534 (61.4)	777 (89.3)
Level III	328 (37.7)	190 (21.8)	518 (59.5)
Level IVa	156 (17.9)	30 (3.4)	186 (21.4)
Level IVb	50 (5.7)	4 (0.5)	54 (6.2)
Level IV	156 (17.9)	30 (3.4)	186 (21.4)
Level Va	211 (24.3)	55 (6.3)	266 (30.6)
Level Vb	66 (7.6)	11 (1.3)	77 (8.9)
Level Vc	8 (0.9)	2 (0.2)	10 (1.1)
Level VI	1 (0.1)	0 (0.0)	1 (0.1)
Level VIIa	342 (39.3)	345 (39.7)	687 (80.0)
Level VIIb	43 (4.9)	6 (0.7)	49 (5.6)
Level VIII	44 (5.1)	4 (0.5)	48 (5.5)
Level IX	3 (0.3)	0 (0.0)	3 (0.3)
Level X	2 (0.2)	0 (0.0)	2 (0.2)

Abbreviations: LNs, lymph nodes; NPC, nasopharyngeal carcinoma.

### 3.3 Distribution of metastatic LNs in each station

Of the whole cohort, the rates of LNs metastasis in Station 1st, 2nd, and 3rd were 820/870 (94.3%), 532/870 (61.1%), and 199/870 (22.9%), respectively (Table 3). Among 820 patients with a Station 1st metastasis, 203/820 (24.8%) patients exhibited unilateral metastasis, and 617/820 (75.2%) patients displayed bilateral metastasis. There were 329/532 (61.8%) patients with unilateral metastasis and 203/532 (38.2%) patients with bilateral metastasis among 532 patients with Station 2nd metastasis. Moreover, among 199 patients with Station 3rd involvement, the rates of LNs metastasis in the unilateral and bilateral neck were 162/199 (81.4%) and 37/199 (18.6%), respectively.

**TABLE 3:** Distribution of LNs metastasis in each station in 870 NPC patients.

Variables	Unilateral (No. %)	Bilateral (No. %)	Total (No. %)
Station 1st			
Level VIIa and II	203 (23.2)	617 (70.9)	820 (94.3)
Station 2nd			
Level III and Va	329 (37.8)	203 (23.3)	532 (61.1)
Station 3rd			
Level IV, Vb, and Vc	162 (18.6)	37 (4.3)	199 (22.9)

Abbreviations: LNs, lymph nodes; NPC, nasopharyngeal carcinoma.

Additionally, among 203 patients who had unilateral Station 1st LNs metastasis, 86/203 (42.4%) and 37/203 (18.2%) patients exhibited ipsilateral Station 2nd and 3rd metastasis, respectively, and 3/203 (1.5%) and 1/203 (0.5%) patients revealed contralateral Station 2nd and 3rd LNs metastasis, respectively. Among the 617 patients who had bilateral Station 1st LNs involvement, the rates of Station 2nd and Station 3rd metastasis increased to 445/617 (72.1%) and 162/617 (26.3%), respectively.

### 3.4 Skipping metastasis

**FIGURE 1:**
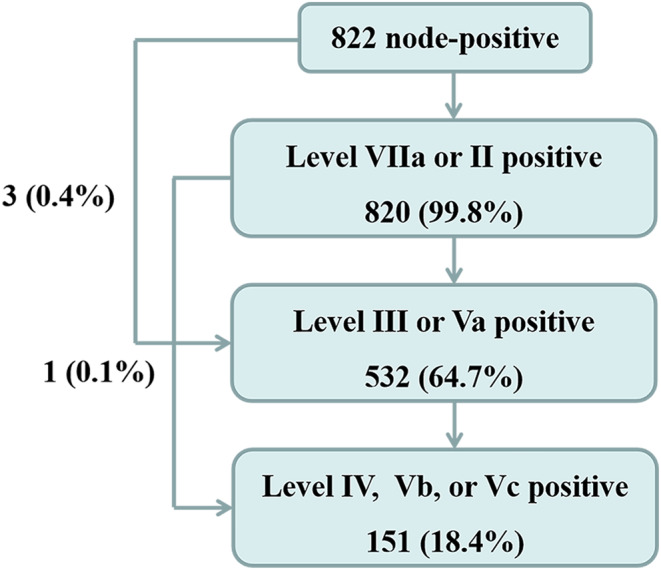
Distribution of metastatic lymph nodes. Station 1st includes level VIIa and II, Station 2nd includes level III and Va, and Station 3rd includes level IV, Vb, and Vc.

Only four patients were regarded as having skipping metastasis (4/870, 0.5%) (Figure 1). Among these four patients, three had Station 2nd metastasis without Station 1st metastasis, and one patient had Station 3rd metastasis without Station 2nd metastasis.

### 3.5 LNs metastasis in extra‐regional areas

Among the 870 patients, only two (0.2%) patients had level Ia metastasis, with one staged T4N3M0 and another staged T3N3M0. The rate of level Ib metastasis was 67/870 (7.7%). All of the 67 patients exhibited level II metastasis, and 59 patients revealed level III metastasis. Among the 67 patients, 3/67 (4.5%) cases staged as N1, 29/67 (43.3%) cases staged as N2, and 36/67 (53.7%) cases staged as N3. A total of 49 patients displayed level VIIb metastasis, and 48 patients had level VIII metastasis. All of the 48 patients with level VIII metastasis had level II involvement, including 2 cases of N1 stage, 16 cases of N2 stage, and 30 cases of N3 stage, and the two patients with N1 stage had the local disease staged T4. Of the entire cohort, the LNs metastasis rate in level IX was 3/870 (0.3%), including one case of N3 stage and two cases of N2 stage. Besides, 2/870 (0.2%) patients had level X metastasis, including 1 case of N3 stage and another of N2 stage.

## 4 DISCUSSION

Metastatic LNs distribution patterns in NPC have been widely discussed. The study by Tang et al. demonstrated that the RLNs and level II represented the first echelon of LNs spread, followed by level III and V, and level IV and supraclavicular fossa (SCF).^20^ Similarly, the study by Li and colleagues categorized the lymphatic drainage of NPC into the upper neck (RLNs and level II), middle neck (levels III and Va), and lower neck (levels IV, Vb, and SCF), and observed successively decreased LNs distribution among the upper, middle, and lower neck.^22^ Based on previous investigations and changes in the 2013 guidelines compared with the 2003 consensus,^26^ the present study categorized NPC lymphatic drainage into intra‐regional and extra‐regional areas, and further divided the intra‐regional areas into three stations: Station 1st (level VIIa and II), Station 2nd (level III and Va), and Station 3rd (level IV, Vb, and Vc) according to the 2013 guidelines.^23^ Our results indicated that the spread patterns of LNs were consistent with which of previous studies, including common LNs involvement (822/870, 94.5%), a high incidence of bilateral metastasis (624/870, 71.7%), and an orderly manner of LNs spread from Station 1st to 3rd with rare skipping metastasis (4/870, 0.5%).

**FIGURE 2:**
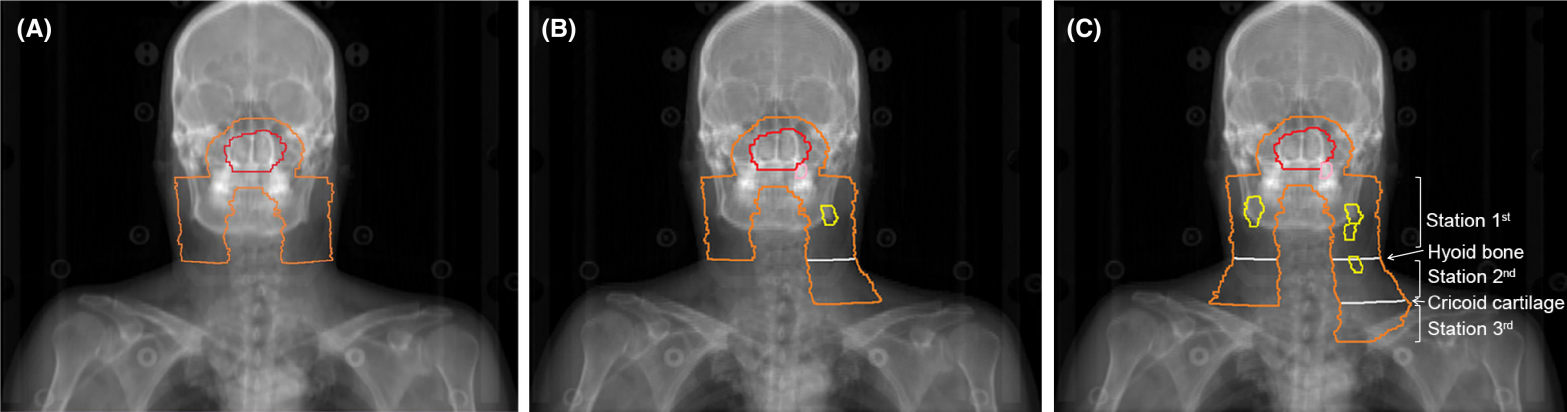
Examples and schematic diagram of individualized elective prophylactic neck irradiation. (A) In node‐negative nasopharyngeal carcinoma (NPC), only bilateral Station 1st is required to be prophylactically irradiated. (B) In NPC with unilateral lymph nodes (LNs) metastasis (Station 1st only in this example), prophylactical irradiation to ipsilateral latter node‐negative station (Station 2nd) and contralateral Station 1st is required. (C) In NPC with bilateral LNs metastasis (Station 1st and Station 2nd involved on the right side and only Station 1st involved on the left side), prophylactical irradiation to ipsilateral latter nodenegative station (irradiation to Station 3rd on the right side and Station 2nd on the left side) is required. The red, pink, yellow, orange, and white lines represent the primary gross tumor, the metastatic retropharyngeal LNs, the metastatic neck nodes, the prophylactically irradiated areas, and the boundaries among the three stations, respectively.

The prognosis of NPC has significantly improved over the past couple of decades,^27^ and the late toxicities following irradiation are commonly observed in NPC patients, even in those treated with intensity‐modulated radiation therapy (IMRT).^28^ Thus, optimizing clinical tumor volume (CTV) is required. Although UNI is recommended in patients with N0 stage disease or only RLN‐positive disease, patients with neck node‐positive disease are still recommended to receive WNI.^18^ The most recent phase 3 trial revealed that UNI would be a valid option to consider even for N1‐stage NPC, and proposed that UNI for patients with ipsilateral N3 disease remains to be evaluated.^8^ However, the phase 3 trial still divided the lymphatic drainage area into upper neck and lower neck. In the current study, we divided the lymphatic drainage area into three stations. The rate of skipping metastasis was only 0.5% among the three stations, and similar results were reported by Li et al.^22^ This orderly spread pattern represents the theoretical foundation of the iEPNI, defined as prophylactical irradiation to the ipsilateral latter node‐negative station in NPC. A detailed explanation of iEPNI is proposed as follows: irradiation to Station 1st in patients who do not have LNs metastasis, irradiation to Station 2nd in patients who only have Station 1st metastasis, and irradiation to Station 3rd in patients who have Station 2nd metastasis but do not have Station 3rd metastasis (Figure 2).

The nasopharynx is a middle structure with vast lymph capillary network drainage into bilateral neck LNs. Although unilateral LNs metastasis occurs less frequently than bilateral metastasis in NPC, neck metastatic LNs are primarily distributed down to ipsilateral lower lymphatic drainage with rare cross‐drainage between bilateral neck nodes. Similarly, our results revealed that NPC with unilateral Station 1st involvement tended to spread down to ipsilateral lymphatic drainage to Station 2nd (42.4%) and Station 3rd (18.2%) rather than contralateral Station 2nd (1.5%) and Station 3rd (0.5%). A recent prospective study reported non‐inferiority survival outcomes of contralateral UNI compared with bilateral WNI in patients with unilateral LNs metastasis.^15^ Furthermore, a retrospective analysis of LNs involvement patterns in 167 NPC indicated that contralateral lymphatic drainage other than RLNs and level II could be spared in unilateral NPC.^29^ Based on previous studies and our results, we propose that it might be feasible to prophylactically irradiate the contralateral Station 1st in NPC patients with unilateral LNs metastasis.

Few studies focused on LNs involvement in extra‐regional areas before the 2013 guidelines were implemented. In the present study, the rates of LNs metastasis in extra‐regional areas were 2/870 (0.2%) for level Ia, 67/870 (7.7%) for level Ib, 1/870 (0.1%) for level VI, 49/870 (5.6%) for level VIIb, 48/870 (5.5%) for level VIII, 3/870 (0.3%) for level IX, and 2/870 (0.2%) for level X based on the 2013 guidelines.^23^ Similarly, Wang et al. provided a detailed description of the distribution of LNs in extra‐regional areas as: 0 in level Ia and VI, 115 (4.3%) in level Ib, 178 (6.6%) in level VIIb, 53 (2.0%) in level VIII, 2 (0.1%) in level IX and Xa, and 3 (0.1%) in level Xb.^19^ Moreover, Jiang and colleagues observed no LNs involved in levels Ia, VI, IX, and X, and reported rates of LNs metastasis of 5.41%, 1.04%, and 0.72% in levels Ib, VIII, and VIIb, respectively.^21^ LNs in extra‐regional areas do not directly receive lymphatics from the nasopharynx, and such involvement may result from the reflux of lymphatics due to a blockage of routine drainage pathways or the tumor invasion of adjacent structures that have direct lymphatic drainage into the extra‐regional areas.^23^ Since the rate of LNs metastasis in level Ib is relatively high compared with other extra‐regional levels, the prophylactical irradiation of level Ib based on the 2018 guidelines^18^ is still required, and the irradiation of extra‐regional areas, except for level Ib, must be considered when LNs involvement occurs.

Our study has several limitations. First, the status of metastatic LNs, including the diameters, numbers, necrosis, and extranodal extension, was not included in the analysis, because the variables were difficult to define only based on MRI documents. Second, since the included patients were treated between January 2019 and December 2019, survival data was limited. Therefore, a further prospective study of prophylactical irradiation based on the three‐station categorization of lymphatic drainage is in preparation. However, our study still provides a clue of iEPNI strategy, which may spare the irradiation of level III and Va in patients who do not have LNs metastasis, and spare the irradiation of level IV, Vb and Vc in patients who only have Station 1st metastasis.

## 5 CONCLUSIONS

In summary, the incidence of LNs metastasis appears high in NPC, and the LNs distribution followed the rule from Station 1st down to Station 3rd with rare skipping metastasis. LNs involvement in extra‐regional areas is uncommon, except for level Ib. iEPNI of prophylactical irradiation to the ipsilateral latter node‐negative station is potentially feasible, and prospective studies are warranted to validate this strategy.

## AUTHOR CONTRIBUTIONS

**Lei Wang:** Conceptualization (equal); formal analysis (equal); funding acquisition (equal); investigation (equal); methodology (equal); writing – original draft (equal). **Zheng Wu:** Conceptualization (equal); funding acquisition (equal); methodology (equal); project administration (equal); resources (equal); writing – original draft (equal). **Qian He:** Investigation (equal); software (equal). **Yiting Li:** Data curation (equal). **Subin Wang:** Validation (equal). **Feiping Li:** Resources (equal). **Hui Wang:** Funding acquisition (equal); writing – review and editing (equal). **Wenhui Li:** Conceptualization (equal); supervision (equal); writing – review and editing (equal). **Yaqian Han:** Conceptualization (equal); funding acquisition (equal); supervision (equal); writing – review and editing (equal).

## FUNDING INFORMATION

This study was supported by Hunan Provincial Health Commission Scientific Research Project (202109031170), Hunan Cancer Hospital Climb Plan (2020QH006, ZX2020001), Hunan Province Key R & D Fund (2022SK2051), and Scientific Research Fund Project of Yunnan Education Department (2023Y0662).

## CONFLICT OF INTEREST STATEMENT

Not applicable.

## ETHICS STATEMENT AND CONSENT TO PARTICIPATE

The study adhered to the Declaration of Helsinki and was approved by the Ethics Committee of The Hunan Cancer Hospital (No. KYJJ‐2020‐189). Written consent was waived, and oral consent was obtained via telephone.

## CONSENT FOR PUBLICATION

Not applicable.

## Data Availability

The research data have been deposited in an institutional repository and are available upon request to the corresponding author.
